# Impact of Women Obesity and Obesity Severity on Live Birth Rate after In Vitro Fertilization

**DOI:** 10.3390/jcm9082414

**Published:** 2020-07-28

**Authors:** Cécile Brunet, Safa Aouinti, Fanchon Huguet, Valérie Macioce, Noémie Ranisavljevic, Anna Gala, Antoine Avignon, Thibault Mura, Ariane Sultan

**Affiliations:** 1 Reproductive Medicine Unit, Univ Montpellier, CHU Montpellier, 34295 Montpellier, France; c-brunet@chu-montpellier.fr (C.B.); n-ranisavljevic@chu-montpellier.fr (N.R.); 2 Clinical Research and Epidemiology Unit, Univ Montpellier, CHU Montpellier, 34295 Montpellier, France; s-aouinti@chu-montpellier.fr (S.A.); v-macioce@chu-montpellier.fr (V.M.); 3 Nutrition Endocrinology Diabetes Department, Univ Montpellier, CHU Montpellier, 34295 Montpellier, France; fanchon.huguet@hotmail.com (F.H.); a-avignon@chu-montpellier.fr (A.A.); 4 Reproductive Biology Unit, Univ Montpellier, CHU Montpellier, 34295 Montpellier, France; a-gala@chu-montpellier.fr; 5 PhyMedExp, INSERM, CNRS UMR, University of Montpellier, CHRU Montpellier, 34295 Montpellier, France; 6 Department of Biostatistics, Epidemiology and Public Health, Univ Montpellier, CHU Nimes, 30029 Nimes, France; thibault-mura@chu-nimes.fr

**Keywords:** assisted reproductive technology, in vitro fertilization, live birth, obesity, body mass index, controlled ovarian stimulation

## Abstract

Access to in vitro fertilization (IVF) for obese women varies across centers, and the impact of obesity on IVF outcomes is widely discussed. We assessed the impact of obesity and its severity on live birth rate (LBR) after IVF. We included women treated for IVF in our center. Data were prospectively collected in the BabySentry^TM^ software. LBR per cycle and cumulative LBR including all attempts of the couple were calculated, considering transfer of both fresh and frozen embryos. Of 1588 included women (2379 controlled ovarian stimulations), 70.2%, 19.5%, 7.9%, and 2.4% were normal-weight, overweight, class I obesity, and class II/III obesity, respectively. For each cycle, LBR did not differ according to BMI category. Adjusted odds ratios (95% confidence intervals) for obtaining a live birth at the first cycle were 1.11 (0.78–1.58) for overweight, 1.17 (0.70–1.95) for class I obese, and 1.05 (0.48–2.31) for class II/III obese women, as compared with normal-weight women. Similarly, no significant associations were found at cycles 2, 3, and 4. Cumulative LBR increased with the number of cycles, independently of the BMI class (*p* log-rank = 0.91). After adjustment, obesity status did not impact significantly the miscarriage rate, regardless of the cycle. In conclusion, neither women obesity nor its severity impacted the cumulative LBR after IVF.

## 1. Introduction

Obesity, a major problem of public health, intervenes at different levels on the reproductive function. It is associated with increased conception time [1] and decreased fecundity [2]. Although the number of obese women in demand of assisted reproductive technology (ART) is unknown, this is a common situation in clinical practice.

According to the NICE guidance on fertility problems, women have to be informed that BMI should ideally be in the range 19–30 kg/m^2^ before starting assisted reproduction, and that a BMI outside this range is likely to reduce ART procedures success [3]. A BMI greater than 35 kg/m^2^ is often considered as a contraindication to ART [4,5], and many centers around the world are denying access to ART program for obese women, in particular in vitro fertilization (IVF). In France, there is no legislation and each center has its own practices.

Beyond access to IVF, the impact of obesity on oocytes number, clinical pregnancy rate, and live birth rate is also widely discussed [6,7,8,9]. Most studies investigating the impact of obesity on IVF results have not specifically assessed the impact of obesity severity. Given the limited access of obese women to IVF, few studies included women with a BMI greater than 35 kg/m^2^. Most studies considered only fresh embryo transfer [6,10,11,12,13] or only the first stimulation attempt [9,14]. Thus, the impact of obesity on IVF results remains unclear, in particular according to the grade of obesity, and studies taking into account both the entire embryonic cohort (fresh and frozen or vitrified embryos) and all stimulation attempts are lacking.

Therefore, this study aimed to assess the impact of obesity and obesity severity on the live birth rate after IVF+/-ICSI in a French cohort. We considered both the live birth rate per cycle and the cumulative live birth rate including all the attempts of the couple.

## 2. Experimental Section

### 2.1. Population and Design

This retrospective monocentric cohort study was conducted in the Reproductive Medicine Unit of Montpellier University Hospital between April 2011 and December 2017, among women treated for IVF (+/-ICSI). In this center, BMI category is not an exclusion criteria for access to IVF. We excluded from our analyses non-nulliparous women in the current couple, those undergoing IVF for pre-implantation genetic diagnosis, those for whom cycles were cancelled before oocyte pickup, those who benefited from 4 stimulations cycles in other centers, and those with BMI < 18.5 kg/m^2^.

### 2.2. IVF Procedure

We chose agonist or antagonist protocol for controlled ovarian stimulation (COS) according to the infertility cause. Gonadotrophins used were r-FSH or HMG according to the woman hormonal profile with a starting dose adapted to age, ovarian reserve and BMI. Oocyte pick-up was performed 36 h after triggering with HCG when at least 3 follicles reached 17 mm. Embryo transfer was performed with ultrasonoguidance at day 3 or 5 after oocyte pick-up. Luteal support was obtained with vaginal progesterone at 600 mg/day starting on the oocyte pick-up day until the βHCG (β-human chorionic gonadotropin) test day. Clinical pregnancy was confirmed with transvaginal ultrasonography at 6 gestational weeks after two positive βHCG tests. For frozen embryo transfer, women were prepared with a natural cycle when they were ovulatory and with a substituted cycle when they were not. The substituted cycle was obtained with oral natural estrogen and vaginal progesterone was introduced when endometrium thickness reached 7 mm.

### 2.3. Data Collection

All data were prospectively collected in the BabySentryTM software (Babysentry Ltd., San Pedro, CA, United States), a central database electronic medical record. For each IVF initiated, we collected (i) basic patients characteristics including BMI, infertility etiology, baseline ovarian reserve parameters at day 3 of the cycle, COS parameters, (ii) stimulation results (number of oocytes obtained, number of embryos obtained on second day), (iii) results of transfer (number of embryo(s) transferred, day of transfer, number of frozen embryo(s)) (iv) modality of the attempt (IVF or ICSI) and (v) outcome of IVF (live birth or miscarriage). Cycle 1 was defined as the first IVF cycle that the patient starts in our center, cycle 2 as the second cycle that the patient starts in our center, and so on. Primary outcome was the live birth rate.

### 2.4. Statistical Analysis

Continuous variables were described with means and standard deviation or median and interquartile range. Categorical variables were described with frequencies. Missing data were not taken into account to calculate frequencies. Women were divided into five groups according to the World Health Organization (WHO) classification cut-points: normal-weight (18.5–24.9 kg/m^2^), overweight (25–29.9 kg/m^2^), class I obesity (30–34.9 kg/m^2^), class II obesity (BMI 35–39.9 kg/m^2^) and class III obesity (BMI ≥ 40 kg/m^2^). Class II and III obesity were gathered due to the small number of women. Normal-weight women were the reference group.

Patients’ characteristics and ovarian simulation methods were compared according to BMI category using ANOVA or Kruskal–Wallis test for quantitative variables, and with chi-square analysis or Fisher’s exact test for categorical variables. To assess the impact of BMI on live birth rate or occurrence of miscarriage, logistic regressions were used to calculate raw and adjusted odds ratios (OR) along with their 95% confidence intervals (CI). OR were adjusted on potential confounding factors: Age, smoking status, infertility causes and anti-Müllerian hormone (AMH) plasmatic level.

The cumulative live birth incidence curve according to the BMI class was constructed with Kaplan–Meier curve using the cycle as unit of time. Women who were not pregnant in a cycle and did not carry out other stimulation cycle later were censored from this point. Women who were followed-up in our center only from the 2nd, 3rd, or 4th cycle (without live birth resulting from previous cycles) were entered into the analysis using truncation method on the left. The curves were compared between the 4 BMI classes using a log-rank test. Statistical bilateral significance threshold was set at 5%. All statistical analyses were performed using SAS^®^ Enterprise Guide (SAS Institute, Cary, NC, USA) version 7.12.

## 3. Results

### 3.1. Population Characteristics

**Figure 1:**
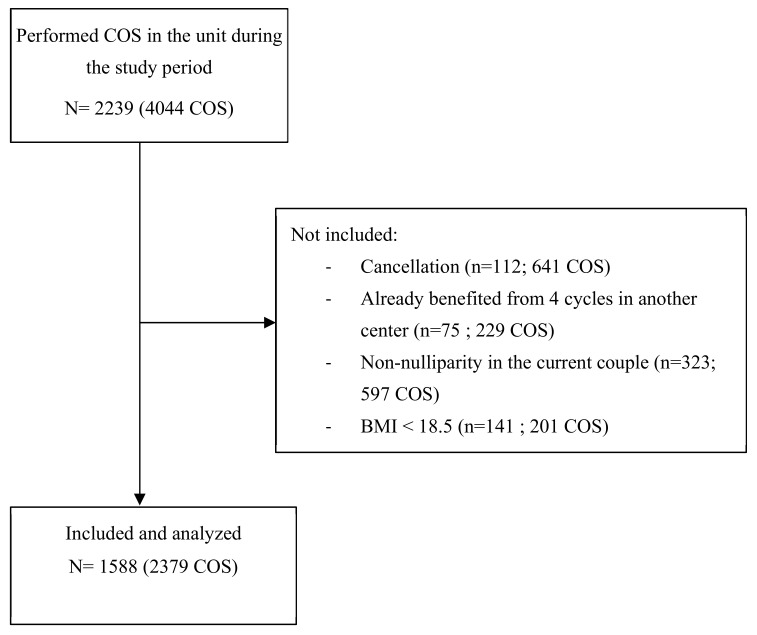
Flow chart. COS: controlled ovarian stimulation.

Of 2239 women treated in the study center, representing 4044 COS, 1588 eligible patients (1279 COS) were included (Figure 1). Among them, 1115 had a normal BMI (70.2%), 309 were overweight (19.5%), and 164 were obese (10.3%). Of these, 126 women had class I obesity (77%) and 38 had grade II/III obesity (23%). Table 1 shows women characteristics according to BMI category. Mean age was similar between subgroups of women. Distribution of infertility etiology was significantly different regarding BMI, with a higher prevalence of dysovulation and polycystic ovarian syndrome (PCOS) among obese women.

### 3.2. COS Characteristics

Characteristics and results of COS for cycle 1 and 2 according to BMI classes are presented in Table 2. The protocol used for COS was similar whatever the BMI. However, both starting and total dose of gonadotrophins used for stimulation were significantly higher in obese than in normal-weight women, for both cycle 1 and cycle 2. Regarding results of cycles 1 and 2, number of either oocytes collected or mature oocytes or embryos obtained at day 2 was comparable across all BMI classes. However, during the first cycle, the number of fresh embryos transferred significantly decreases with increasing BMI classes. Further, during the second cycle, the total number of transferred embryos was lower for higher BMI classes.

Results for cycle 3 and 4 showed no difference.

### 3.3. Impact of Obesity on Live Birth Rate Per Cycle and on Cumulative Live Birth Rate

**Figure 2:**
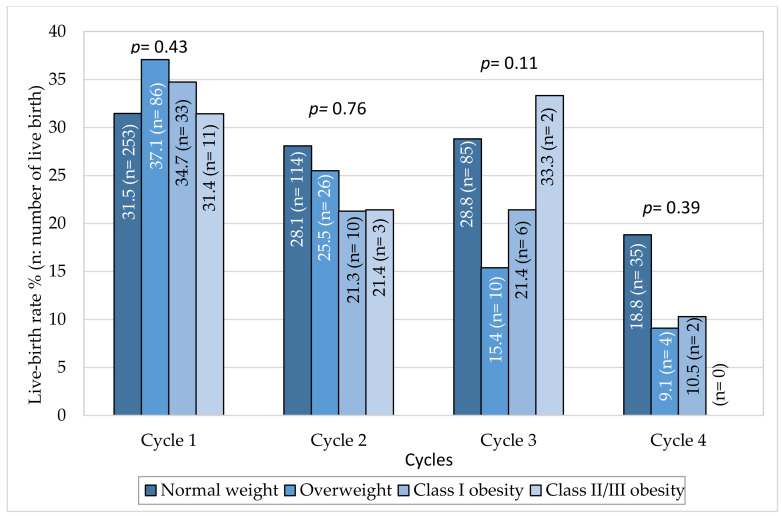
Live-birth rate by cycle and BMI class.

For each cycle, the live birth rate did not differ according to BMI category (Figure 2). During the first cycle, the live birth rate/cycle was 34.7% in class I obese women, and 31.4% in class II/III vs. 31.5% in normal-weight women. In the second cycle, the live birth rate tended to be lower than in the first cycle in all BMI categories.

Adjusted OR for obtaining a first live birth by BMI class are shown in Table 3. After adjustment for age, smoking status, infertility causes and AMH plasmatic levels, neither obesity nor obesity severity affected significantly the chance to obtain a live birth, regardless of the cycle. When considering all women with BMI ≥ 25 kg/m^2^, their chance to obtain a live birth was not significantly different from that of normal-weight women for each cycle, and overall for the 4 cycles (adjusted OR 0.90, CI 95% 0.72–1.13, *p* = 0.36).

**Figure 3:**
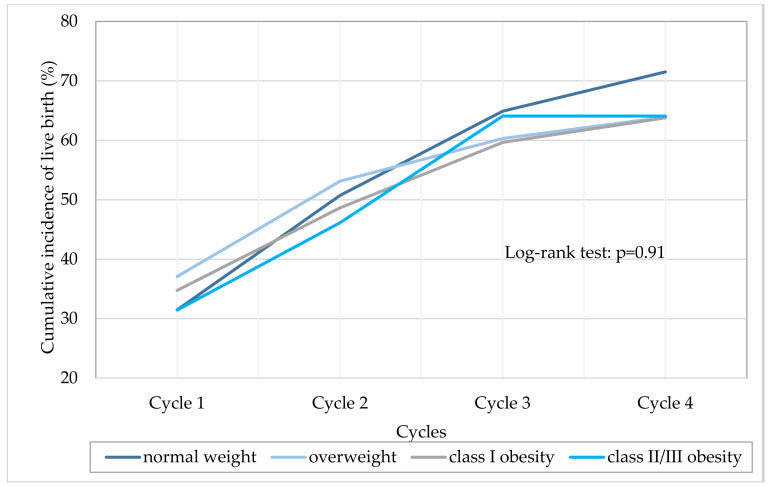
Cumulative incidence of live birth by BMI class.

The cumulative live birth rate, defined as live birth rate at the end of the attempts, was represented for each BMI class (Figure 3). Cumulative live birth rate increased with the number of cycles, independently of BMI (*p* log-rank = 0.91).

### 3.4. Impact of Obesity on Rank of Transfer, Fresh, or Frozen Embryo Transfer and Miscarriage

For each cycle, live birth was mainly obtained after the first transfer, regardless of BMI. Indeed, median rank of embryo transfer to obtain a live birth was 1 for all cycles and all BMI classes (Table S1). Further, percentages of women with at least one fresh and frozen embryo transfer that resulted in live birth at each cycle were not significantly different according to BMI category. At cycle 1, respectively 20.5%, 22.8%, 28.4%, and 17.4% of women with at least one fresh embryo transfers (*p* = 0.29) and 13.8%, 18.4%, 8.8%, and 17.2% of women with at least one frozen embryo transfers (*p* = 0.21) obtained a live birth for normal-weight, overweight, class I obese and class II/III obese women, respectively (*p* = 0.29) (Table S2).

Finally, we expressed the OR of miscarriage at the first clinical pregnancy for each BMI class compared to normal-weight women. BMI did not impact significantly the miscarriage rate, regardless of the cycle and after adjusting for confounding factors (age, smoking habits, infertility causes, and AMH plasmatic level) (Table S3).

## 4. Discussion

This large cohort study showed that neither women obesity nor its severity affected IVF outcomes. Its originality lies in taking into account all stimulation attempts and both fresh and frozen embryos, whereas most studies considered only the first cycle or only fresh embryos [15]. We also assessed the rank of the transfer that allowed the live birth for each BMI class.

We found that neither obesity nor obesity severity do affect significantly the live birth rate regardless of the cycle, both on raw data and after adjustment for potential confounding factors. Indeed, cumulative live birth rate increased with the number of cycles, independently of BMI. Further, we showed that the live birth rate was higher during the first cycle of stimulation in all BMI classes. Despite fewer embryos transferred with increasing BMI, live birth at each cycle was mainly obtained after the first embryo transfer, regardless of BMI.

Data from the literature are not consensual regarding the effect of obesity and its severity on live birth rate. Indeed, some studies did not find any deleterious effect of BMI, with no significant difference in the live birth rate between normal-weight and overweight women [16,17], but also between normal-weight and obese women [7,18] and between BMI categories [19]. Likewise, Coyne et al. showed that gestational carriers’ BMI does not impair reproductive outcomes including live birth rate [20]. On the contrary, other studies found a negative impact of obesity and of each of the grades of obesity on the live birth rate compared to normal BMI [6,9,21], specifically in women under 35 years [22], and for obesity in general compared to reference BMI category [12]. Recently, a meta-analysis concluded that live birth rate was significantly decreased in obese women compared with normal-weight women, with a relative risk of 0.85 (CI 95%: 0.84–0.87) [15]. Differences in study populations might explain such controversial results, as most published studies were conducted in the US, with different inclusion criteria as regards age and PCOS status. Particularly, results of the latter meta-analysis were largely influenced by two US studies with very large sample size including only fresh embryos [6,12]. Moreover, the reported loss of chance of 15% yields an absolute chance of giving birth that is acceptable for patients and does not justify refusing access to IVF to obese women. This supports the conclusions of Tremellen et al. that prohibiting obese women’s access to IVF may be unfair, as live birth rate in obese women may be far better than that observed for many older women who are allowed access to IVF [23]. In our study, we showed that after 4 cycles, 64% of obese women gave birth to a child. This rate was similar among overweight, class I and class II/III obesity.

Similarly, the impact of obesity on clinical pregnancy rate is also debated. A meta-analysis showed that clinical pregnancy rate significantly decreased by 13% in obese women compared to normal-weight women, with however significant heterogeneity in the included studies [24]. On the contrary, other studies found no impact of obesity on clinical pregnancy [7,25,26]. It is therefore difficult to be consensual regarding the impact of obesity on IVF prognosis.

On the other hand, weight loss interventions based on nutritional and physical activity changes generally lead to limited weight loss. The impact of most dietary interventions is short-lived and the weight lost is often taken up over time. A randomized controlled trial showed that in women with BMI between 30 and 35 kg/m^2^, weight loss did not improve the live birth rate either in the first cycle or after two years, and that regain of pre-study weight occurred in most women of the weight-loss group [27,28]. Therefore, these interventions may not only be insufficient but also take some time to allow these obese women to cross BMI thresholds. Further, while age is the main predictor of ARTS success [29], preventing obese women from accessing them can cost them valuable time thus diminishing their chances of conceiving. As regards bariatric surgery, it may be associated with higher risks of small-for-gestational-age infants, shorter length of gestation, and potentially increased risk of stillbirth or neonatal death [30]. Finally, allowing or limiting fertility treatment based on BMI leads to feelings of stigma and injustice, which may in turn sustain failure to lose weight [31]. However, this does not mean that weight loss should not be encouraged.

Further research is required to determine the number of obese women in demand of ART, and to examine the impact of women obesity and its severity on IVF outcomes in larger multicentric cohorts.

Our work has some limitations, based on a retrospective study with data from register. Some women have to be considered as lost to follow-up, as we do not have any information after the IVF failure. In addition, excluding women with cancelled cycles may have induced selection bias as the proportion of cycle cancellations may differ according to the BMI. Further, women with BMI above 35 kg/m^2^ and notably above 40 kg/m^2^ were too few to allow analysis regarding grade III obesity. Moreover, we cannot assume that this obese women sample has the same profile as obese women of childbearing age in France, as we do not have any data regarding obese infertile women that did not consult nor for the ones that could not attempt for ART. In particular, some obese women might have been discouraged from attempting ART, even though there is no exclusion criteria based on BMI in our center. One of the main limits of this study is the lack of data regarding complications during pregnancy, as it is well demonstrated that obesity is a major risk factor for gestational complications [32].

## 5. Conclusions

Our study showed that women obesity does not impact the cumulative live birth rate after IVF. Therefore, we do not recommend prohibiting access to ART for obese women. The decision of ART must be integrated into a shared medical decision, while warning women of risks related to obesity.

## Figures and Tables

**Table 1:** Characteristics of Women Included in the Study According to Their BMI Class.

Variables	Normal Weight(*n* = 1115)	Overweight(*n* = 309)	Class I Obesity(*n* = 126)	Class II/III Obesity(*n* = 38)	*p*-Value
Age (years)	33.3 ± 4.9	32.6 ± 5.6	33.5 ± 5.7	32.7 ± 4.8	0.32
Smoking (%)	26.2	21.2	22.7	20	0.28
Infertility etiology					
Dysovulation (%)	7.5	16.8	16.7	31.6	<0.001
Tubal factor (%)	14.1	15.5	16.7	18.4	0.72
Endometriosis (%)	21.6	15.9	8.7	7.9	<0.001
PCOS (%)	6.6	13.3	15.9	21	<0.001
Male infertility (%)	55.7	57.3	63.5	65.8	0.25
Idiopathic (%)	13.5	9.1	10.3	0	0.015
Other (%)	1.5	1.9	1.6	0	0.91
Gestity and parity					
Gestity	0.48 ± 0.98	0.59 ± 1.06	0.57 ± 1.00	0.53 ± 1.2	0.26
Parity	0.10 ± 0.44	0.11 ± 0.43	0.17 ± 0.57	0.05 ± 0.23	0.24
Ovarian reserve					
AMH	3.6 ± 3.4	4.1 ± 4.5	3.4 ± 2.4	4.7 ± 3.8	0.005
FSH	7.5 ± 4.5	7.0 ± 4.3	6.6 ± 1.7	5.9 ± 1.6	<0.001

Data are means ± standard deviations, or percentages. PCOS: polycystic ovarian syndrome; AMH: anti-Müllerian hormone; FSH: Follicle-stimulating hormone.

**Table 2:** Characteristics of Controlled Ovarian Stimulation for the Two First Cycles by BMI Class.

	Cycle 1					Cycle 2				
	Normal Weight(*n* = 804)	Over-Weight(*n* = 232)	Class I Obesity(*n* = 95)	Class II/III Obesity(*n* = 35)	*p*- Value	Normal Weight(*n* = 406)	Over-Weight(*n* = 102)	Class I Obesity(*n* = 47)	Class II/III Obesity(*n* = 14)	*p*-Value
Protocol (%)										
Agonist	44.7	50.4	45.3	40	0.41	37.9	42.2	44.7	42.9	0.73
Antagonist	55.3	49.6	54.7	60		62.1	57.8	55.3	57.1	
ART technique (%)										
IVF	40.8	40.7	39.8	25	0.36	24.4	23.7	15.6	23.1	0.65
ICSI	59.2	59.3	60.2	75		75.6	76.3	84.4	76.9	
Gonadotrophin										
Starting dose (UI)	206 ± 74	199 ± 72	231 ± 61	236 ± 75	<0.001	226 ± 75	223 ± 76	247 ± 71	261 ± 78	0.19
Total dose (UI)	2138 ± 850	2158 ± 884	2449 ± 774	2624 ± 992	<0.001	2275 ± 871	2307 ± 909	2689 ± 972	3138 ± 1346	0.009
Duration (days)	10.3 ± 1.3	10.6 ± 1.6	10.4 ± 1.4	10.8 ± 1.6	0.05	10.1 ± 1.4	10.2 ± 1.6	10.6 ± 1.9	11.5 ± 2.5	0.009
Number of oocytes collected	10.6 ± 6.0	10.7 ± 5.9	10.9 ± 6.2	10.8 ± 6.1	0.88	9.8 ± 5.7	10.3 ± 5.4	10.7 ± 6.3	8.7 ± 5.0	0.45
Number of mature oocytes	5.1 ± 5.8	5.1 ± 5.4	5.3 ± 5.9	5.9 ± 5.8	0.62	5.7 ± 5.1	6.1 ± 5.5	6.5 ± 4.9	5.6 ± 5.1	0.66
Number of embryos obtained at day 2	5.8 ± 4.4	5.6 ± 3.9	5.0 ± 4.1	5.2 ± 4.7	0.31	5.2 ± 3.9	5.4 ± 4.2	4.0 ± 3.3	5.6 ± 4.7	0.19
Number of fresh transferred embryos	1.2 ± 0.8	1.1 ± 0.8	1.3 ± 0.8	0.9 ± 0.9	0.006	1.4 ± 0.9	1.4 ± 0.9	1.3 ± 0.9	1.2 ± 0.8	0.45
Total number of transferred embryos	1.9 ± 1.2	1.8 ± 1.1	1.8 ± 1.2	1.5 ± 1.1	0.04	2.0 ± 1.3	2.2 ± 1.3	1.6 ± 1.3	1.4 ± 0.8	0.006

Data are means ± standard deviations or percentages; ART: assisted reproductive technique; IVF: in vitro fertilization; ICSI: intracytoplasmic sperm injection.

**Table 3:** Adjusted Odds Ratios for Obtaining a First Live Birth by BMI Class.

	Adjusted OR [95% CI] ^‡^							
BMI Classes	N	Cycle 1	N	Cycle 2	N	Cycle 3	N	Cycle 4
Normal weight	804	1	406	1	295	1	186	1
Overweight	232	1.11 (0.78–1.58)*p* = 0.55	102	0.83 (0.47–1.46)*p* = 0.51	65	0.45 (0.20–1.03)*p* = 0.06	44	0.44 (0.12–1.56)*p* = 0.20
Class I obesity	95	1.17 (0.70–1.95)*p* = 0.55	47	0.68 (0.29–1.55)*p* = 0.36	28	0.97 (0.36–2.64)*p* = 0.95	19	0.59 (0.11–3.11)*p* = 0.54
Class II/III obesity	35	1.05 (0.48–2.31)*p* = 0.90	14	0.69 (0.14–3.37)*p* = 0.65	6	0.87 (0.08–9.39)*p* = 0.91	1	-

^‡^: Odd ratios adjusted by stimulation age, smoking status, infertility etiologies and AMH levels.

## Supplementary Materials

The following are available online at https://www.mdpi.com/2077-0383/9/8/2414/s1, Table S1: Median rank of embryo transfer to obtain a first live birth; Table S2: Percentages of women with at least one fresh and/or frozen embryo transfer that resulted in live birth by cycle and BMI class; Table S3: Adjusted odd ratios for miscarriage at the first clinical pregnancy by cycle and BMI class.
