# Supplementary material for: Impact of Women Obesity and Obesity Severity on Live Birth Rate after In Vitro Fertilization
Source: J Clin Med. 2020 Jul 28;9(8):2414. doi: 10.3390/jcm9082414 (PMC7463979; doi:10.3390/jcm9082414)
Supplement: Supplementary file 1 [file jcm-09-02414-s001.pdf]

Table S1. median rank of embryo transfer to obtain a first live birth.

	Cycle 1		Cycle 2		Cycle 3		Cycle 4	
	**N**	**Median (Q25; Q75)**	**N**	**Median (Q25; Q75)**	**N**	**Median (Q25; Q75)**	**N**	**Median (Q25; Q75)**
Normal weight	247	1 (1; 2)	112	1 (1; 1)	83	1 (1; 1)	34	1 (1; 1)
Overweight	85	1 (1; 2)	25	1 (1; 2)	10	1 (1; 1)	4	1 (1; 1)
Class I obesity	33	1 (1; 1)	10	1 (1; 2)	6	1 (1; 1)	2	1 (1; 1)
Class II/III obesity	10	1 (1; 1)	3	1 (1; 2)	2	1 (1; 1)	0	-
p		0.25		0.20		0.47		0.55

Table S2. Percentages of women with at least one fresh and/or frozen embryo transfer that resulted in live birth by cycle and BMI class.

BMI Classes	Cycle 1		Cycle 2		Cycle 3		Cycle 4	
	Fresh Embryo Transfer	Frozen Embryo Transfer	Fresh Embryo Transfer	Frozen Embryo Transfer	Fresh Embryo Transfer	Frozen Embryo Transfer	Fresh Embryo Transfer	Frozen Embryo Transfer
**Normal weight**	165/804 (20.52 %)	88/639 (13.77 %)	78/406 (19.21 %)	36/328 (10.98 %)	61/295 (20.68 %)	24/234 (10.26 %)	22/186 (11.83 %)	13/164 (7.93 %)
**Overweight**	53/232 (22.84 %)	33/179 (18.44 %)	14/102 (13.73 %)	12/88 (13.64 %)	10/65 (15.38 %)	0/55	3/44 (6.82 %)	1/41 (2.44 %)
**Class I obesity**	27/95 (28.42 %)	6/68 (8.82 %)	8/47 (17.02 %)	2/39 (5.13 %)	5/28 (17.86 %)	1/23 (4.35 %)	2/19 (10.53 %)	0/17
**Class II/III obesity**	6/35 (17.14 %)	5/29 (17.24 %)	3/14 (21.43 %)	0/11	2/6 (33.33 %)	0/4	0/1	0/1
p-value	0.29	0.21	0.59	0.42	0.58	0.04	0.76	0.37

Values are number of live births / number of women with at least one fresh or frozen embryo transfers (%).

Table S3. Adjusted odd ratios for miscarriage at the first clinical pregnancy by cycle and BMI class.

	**Cycle 1 (*n* = 508)**	**Cycle 2 (*n* = 221)**	**Cycle 3 (*n* = 141)**
BMI Class	Adjusted OR ‡ [95% CI]	Adjusted OR ‡ [95% CI]	Adjusted OR ‡ [95% CI]
Normal weight	1	1	1
Overweight	1.03 [0.50–2.09] *P* = 0.934	1.96 [0.79–4.85] *P* = 0.145	0.535 [0.05–5.27] *P* = 0.592
Class I obesity	0.72 [0.25–2.05] *P* = 0.541	2.43 [0.66–8.93] *P* = 0.180	0.838 [0.08–8.31] *P* = 0.880
Class II/III obesity	1.70 [0.42–6.93] *P* = 0.458	0.978 [0.07–13.97] *P* = 0.987	

n: number of first clinical pregnancies; ‡: Odd ratios adjusted by stimulation age, smoking, dysovulation, tubal factor, endometriosis, PCOS, Male infertility and AMH level.
